# P45 Lyme-associated uveitis: Panuveitis and positive Lyme results – is it a masquerade or the real deal?

**DOI:** 10.1093/rap/rkac067.045

**Published:** 2022-09-28

**Authors:** Lisa Bray, Megan Jeffries

**Affiliations:** St Richard's Hospital, Chichester, United Kingdom; St Richard's Hospital, Chichester, United Kingdom

## Abstract

**Introduction/Background:**

A 5 year old boy presented with unilateral panuveitis and on screening tests was lineblot positive for Lyme IgM in the setting of other screening results being negative. He was treated for active Lyme disease with antibiotics and the uveitis treated with steroids. To date, good responses … but is this a Lyme-associated uveitis, or a red herring result which will evolve into more than simple monophasic disease? The differential of Juvenile Idiopathic Arthritis related uveitis remains a concern, ensuring adequate treatment and monitoring in place for disease evolution is crucial.

**Description/Method:**

5 year old boy referred to Ophthalmology with an absent red reflex, anterior chamber cells, no hypopyon, but no clear fundal views in the left eye. The differential diagnosis was autoinflammatory/infective and possibly Juvenile Idiopathic Arthritis. On subsequent review he had iris vascularisation with anterior vitritis. He started maxidex and atropine drops.

On review by paediatric rheumatology he was noted to have one painless lump on the top of his left foot, no other rashes and no arthritis. An isolated small posterior right cervical chain node <0.5cm was palpable. Urine dip was negative. Screening bloods for infection and autoinflammatory disorders were sent as was a dermatology referral. He had been bitten 1-2 years previously by a tick but not recently. He had occasional fevers lasting less than seven days. There was no family history of autoimmune disease.

Results of screening demonstrated a CRP <1.0, connective tissue disease screening and ANCA negative as was HLA B27 and coeliac screening. Rubella IgG was detected, Varicella IgG negative as were Cytomegalovirus, Syphilis and TB testing. He had a normal chest x-ray.

The dermatology team diagnosed him with subcutaneous granulomatous annulare, a benign self limiting condition with no indication for biopsy and no treatment required.

Lyme serology was subsequently reported as IgG/IgM positive with IgG lineblot indeterminate and IgM lineblot positive. We treated in total with an eight week course of initially intravenous ceftriaxone then oral amoxicillin, followed by oral doxycycline.

Increasing anterior chamber flare led to a methylprednisolone pulse with a slow oral wean. He was referred to Great Ormond Street Hospital for specialist review by the Ophthalmology team and was noted to have good improvement at that time which has continued on local reviews. There is not complete resolution, and he has a cataract which may need surgical intervention.

**Discussion/Results:**

Lyme disease is a tick-borne disease caused by the bacteria *Borrelia burgdoferi*. This can manifest as erythema migrans, fever, headaches, myalgia and fatigue with or without lymphadenopathy. Dissemination can cause arthritis, cranial nerve palsies, meningitis, myocarditis or uveitis.

Lyme uveitis is rare but documented. The initial infection can cause inflammation which can trigger an inflammatory cascade. This can continue once acute infection is treated.

Microbiology advised on antibiotics based upon good penetration for central nervous system cover. After the vitritis had been seen in the right eye with deteriorating vision in the left as we came to the conclusion of the amoxicillin treatment, and we were advised to switch to doxycycline for a further 4 weeks. We discussed the intraocular penetration of amoxicillin and doxycycline and wondered if the amoxicillin was not as effective either because it couldn’t cross into the orbit where there was active disease, or that it had eradicated active disease but a secondary inflammatory cascade had been triggered. *Borrelia* is usually sensitive to antibiotic therapy.

Our patient’s course was mildly complicated by developing active varicella post pulse with methylprednisolone and so required a course of aciclovir. He has tolerated the medications and interventions very well, with few side effects to date.

Having not worked up or seen a case of Lyme-associated uveitis this has been a fascinating learning curve, assessing responses, questions about antibiotic choices and anticipating sequelae for our patient who may still need surgery for the developed cataract in the future. His journey is not yet over and we continue our collaborative multicentre care to obtain the best possible outcome for our patient.

**Key learning points/Conclusion:**

Continuing to suspect infection as an association in a unilateral uveitis, especially panuveitis, is key to diagnosis. Lyme associated uveitis is treatable and with good early control of the triggered inflammatory cascades that cause vitritis can lead to full recovery of visual loss with no need for ongoing systemic immune suppression. Some patients in a small case series demonstrated that with intermediate uveitis or keratitis from chronic inflammation there was a degree of permanent visual loss.

Lyme disease is always a diagnostic quandary, due to difficulty in interpreting indeterminate results and giving those a clinical application. IgM and IgG positivity triggers lineblot reactive testing in most centres or can be requested to give a definitive result.

There are NICE guidelines for management of Lyme disease.

Uveitis can be triggered by a long list of infective or autoinflammatory conditions. Initial screening should encompass a range of those to exclude other causative pathology.

Collaborative working across different specialities and centres of excellence has led us to this point in our patient's care. Knowing when to recognise your own limitations in knowledge and to seek expert opinion from a wider team can be an important step in achieving the best outcome for our patients.

We wondered if anyone else here had experience in managing Lyme-associated uveitis and what their outcomes had been?

Once antibiotics completed would anyone consider retesting the lineblot to check for loss of IgM positivity, and IgG creation? Does this add value clinically and when should it be done? Close working with ophthalmology, rheumatology, immunology and general paediatrics has been key getting to this point.

Would there be an appetite for auditing those cases seen across the centres as a multicentre audit with outcomes and case reports for a case series?

